# Synconset Waves and Chains: Spiking Onsets in Synchronous Populations Predict and Are Predicted by Network Structure

**DOI:** 10.1371/journal.pone.0074910

**Published:** 2013-10-08

**Authors:** Mohan Raghavan, Bharadwaj Amrutur, Rishikesh Narayanan, Sujit Kumar Sikdar

**Affiliations:** 1 Department of Electrical Communication Engineering, Indian Institute of Science, Bangalore, Karnataka, India; 2 Molecular Biophysics Unit, Indian Institute of Science, Bangalore, Karnataka, India; SUNY Downstate MC, United States of America

## Abstract

Synfire waves are propagating spike packets in synfire chains, which are feedforward chains embedded in random networks. Although synfire waves have proved to be effective quantification for network activity with clear relations to network structure, their utilities are largely limited to feedforward networks with low background activity. To overcome these shortcomings, we describe a novel generalisation of synfire waves, and define ‘synconset wave’ as a cascade of first spikes within a synchronisation event. Synconset waves would occur in ‘synconset chains’, which are feedforward chains embedded in possibly heavily recurrent networks with heavy background activity. We probed the utility of synconset waves using simulation of single compartment neuron network models with biophysically realistic conductances, and demonstrated that the spread of synconset waves directly follows from the network connectivity matrix and is modulated by top-down inputs and the resultant oscillations. Such synconset profiles lend intuitive insights into network organisation in terms of connection probabilities between various network regions rather than an adjacency matrix. To test this intuition, we develop a Bayesian likelihood function that quantifies the probability that an observed synfire wave was caused by a given network. Further, we demonstrate it's utility in the inverse problem of identifying the network that caused a given synfire wave. This method was effective even in highly subsampled networks where only a small subset of neurons were accessible, thus showing it's utility in experimental estimation of connectomes in real neuronal-networks. Together, we propose synconset chains/waves as an effective framework for understanding the impact of network structure on function, and as a step towards developing physiology-driven network identification methods. Finally, as synconset chains extend the utilities of synfire chains to arbitrary networks, we suggest utilities of our framework to several aspects of network physiology including cell assemblies, population codes, and oscillatory synchrony.

## Introduction

**Figure 1:**
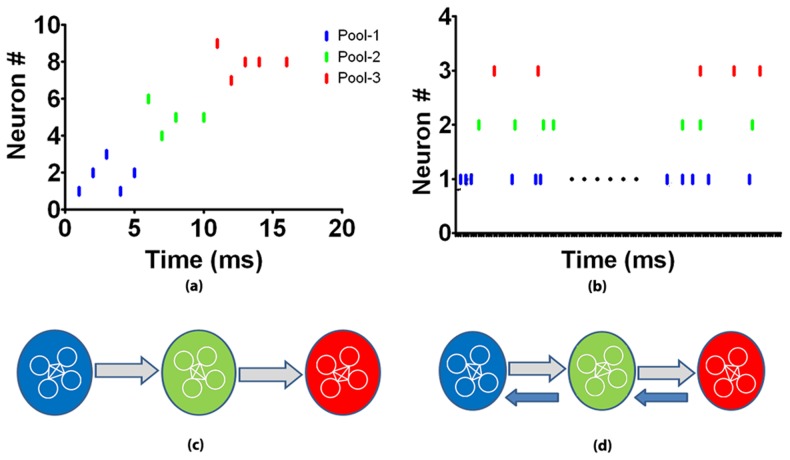
A cartoon illustrating synfire and synconset waves/chains. (**a**) A synfire wave with three pools of neurons resulting from the structure in (c). Note the packets of spikes propagating from pool-1 through pool-3. (**b**) A set of spike trains of three neurons, one from each pool of network in (d). The onsets of the three neurons form a cascade, but there are no spike packets formed. (**c**) A synfire chain with three neuron pools. The block arrows between pools indicate all-to-all connectivity between the neuron pools. (**d**) A synconset chain with three neuron pools. The block arrows between pools indicate all-to-all connectivity between the neuron pools. Note the feedforward chains in grey and the recurrent connections in blue.

During an oscillation, networks of inhibitory neurons selectively suppress different groups of principal cells at different times, moulding principal cells into transiently synchronous ensembles that encode sensory information [1], [2]. At such times the principal cells are known to fire in a phase locked manner with zero lag/lead. While deviations from phase locked firing were thought to be statistical aberrations[3], [4], in the last few years multiple experimental approaches using cultures [5]–[7], slices *in vitro* [8], [9] and behaving animals *in vivo* [10]–[12] have shown that such delays are deterministic. But what is the mechanistic basis of such delays? While it is known that detuning of a synchronous group or conduction delays [13] can cause such millisecond delays, such consistent delays can occur even in the absence of conduction delays and as a direct result of the network architecture. Thus the role of the network architecture in firing sequences of synchronous populations is very important. The presence of synfire chains or feedforward chains of neuron pools [14], [15]) have been proposed to give a network based explanation of such consistent millisecond delays. It has also been proposed that such synfire chain structures may occur in real neuronal networks as embedded chains in a random network [15], [16]. The high utility of synfire chains as a framework for quantifying network structure comes from the elegance of it's relationship with the resultant network activity called synfire waves (Fig. 1a, c). Synfire waves are propagating packets of spikes along the synfire chain and delays between neuron firing times are a direct result of the chain order with upstream pools firing earlier than downstream ones. But synfire chains are limited by the requirement that the background input to the chain be asynchronous or at most mildly correlated with the chain activity [15]. In an arbitrarily connected recurrent network, background activity to neurons is often highly correlated with the feedforward input due to strong recurrent connections from downstream neurons back to upstream neurons thus violating a basic assumption behind synfire chains. As a result analysing these delays and establishing their genesis from the network architecture becomes non-trivial. But it must be noted that while recurrent connections from downstream to upstream neurons can spoil the synfire cascade structure, the first spikes fired by the neurons are essentially due to the feed forward chain. In this context we define the “synconset” wave as the cascade of first spikes as against the cascade of spike packets or synfire waves. We also define a “synconset chain” as a feed forward chain of neuron pools. While synfire chains too are feed forward chains, synfire chains imply that the background input to the chain is asynchronous or at most mildly correlated. But synconset chains make no demands on background activity.(Fig. 1). We now hypothesise that spontaneous formation of synconset chains by stimulation of a part of the network causes synconset waves. In order to verify our hypothesis we first predict the synconset chain that would get activated using the network connectivity structure. The synconset chain trivially predicts a firing order, with upstream pools firing earlier than downstream ones. Now by running network simulations using biophysically realistic conductances in single compartment neurons, we observe the synconset waves. Our hypothesis is verified if the sequence predicted by the synconset chain is observed in the synconset waves.

In the subsequent part of our study, we focussed on the inverse of this problem. Starting from the pattern of delays we ask if something can be said about the network connectivity. In the recent past there has been a significant focus on the study of brain network organisation [17]. Efforts have been made to infer the structural and functional connectivity of brain networks from the level of whole brain networks to small networks of a few hundred or thousand cells [18]–[22]. The typical approach to network reconstruction starting from any dynamic, be it firing rate or delay, consists of estimating a continuous measure of association between pairs of nodes and thresholding the same to yield an adjacency matrix [17]. If the network dynamic under consideration is temporal delay, the characteristic lag between any two neurons is given by the location of the peak in cross correlation histogram. A characteristic delay in the range of a single synaptic delay is taken to imply a connection between the neuron pair [23], [24]. From the perspective of functional organisation, studying pairwise interactions and the consequent synapse level reconstruction is not very helpful as a single pre-synaptic neuron cannot cause the post-synaptic neuron to fire. Information flow in neuronal networks occurs by transmission between groups or pools of neurons. Thus it would be more insightful to reconstruct networks in terms of functional chains over which information is transmitted. Consequently we must start our analysis at the level of firing sequences of multiple neurons. Recognising firing sequences has been achieved in literature using two different approaches. One method pieces together pairwise delays to obtain the firing sequence [11]. The second involves mining for frequent episodes in the spike train data [25], [26]. The former may yield pairwise delays that are not additive(i.e. when measured delays for neuron pair A–C does not equal the sum of delays between pairs A–B and B–C) [11]. In the latter method, associating the firing sequences with the underlying network structures is not easy. In this context we propose that synconset waves could be a good starting point for network reconstruction. Measuring the delays only at the onset [5]–[7] of spiking in each cycle and clustering the patterns obtained is fast and computationally non-intensive. It has been shown that the order of activation within a synchronisation event is non-random, hierarchical [27], [28] and deterministic [8]. It also yields sequences directly instead of pairwise delays. Further, if our hypothesis in the previous section is true, then synconset waves can be associated with an underlying synconset chain. Multiple measured synconset waves uncover multiple synconset chains. Consequently we also propose a representation of the network as an aggregate of several synconset chains or neuron clusters with their connection probability densities. While synconset waves are easily associated with synconset chains, finding the complete network is tougher as multiple networks may contain a given set of synconset chains. In order to simplify the issue we consider a finite set of candidate networks and use a Bayesian framework to identify the candidate network that maximises the likelihood of observing the onset latency patterns obtained from simulation. The candidate network set may consist of representatives from different families of networks and hypothetical networks containing the multiple synconset chains uncovered. Thus instead of network reconstruction the problem is transformed into one of network identification.

## Results

### External stimulation of network causes spontaneous formation of synconset chains and synconset waves provided that the neuron pool sizes are sufficiently large

Every excitatory neuron that has the same set of inhibitory neurons in it's pre-synaptic neuron set can be thought of as having the same graph colour [2]. It has been proposed that the set of neurons with the same graph colour are active or inactive in an oscillation cycle depending on the state of pre synaptic inhibitory neurons [2]. It is within this set that we seek to study the relation between connectivity and latency of activation. We considered the activity in a neuronal network whose dynamics were jointly modulated by external excitatory drive and the resultant oscillatory activity. In our simulated networks, the external drive came from periodic stimulation of a small subset of neurons. Our principal neuron network represented a group of neurons which receive similar inhibitory input and hence shared the same graph colour. For simplicity we use a single inhibition source in our network. The entire network drove and was driven by this global inhibition. Note that inhibition is global within our principal network because we have assumed that the entire network has the same graph colour. In a more general setting one could imagine our principal cell network as being a subnetwork of a larger network, and a common set of inhibitory neurons acting similarly on this subnetwork to either activate or deactivate it during an oscillatory cycle. The external input started a cascade of activity in the network causing feedback inhibition that silenced the network. Inhibition silenced the network and allowed a fresh start of activity in the next cycle. This constituted the oscillatory activity. Thus each cycle afforded a window to the measurement of first spike latencies in the network.

**Figure 2:**
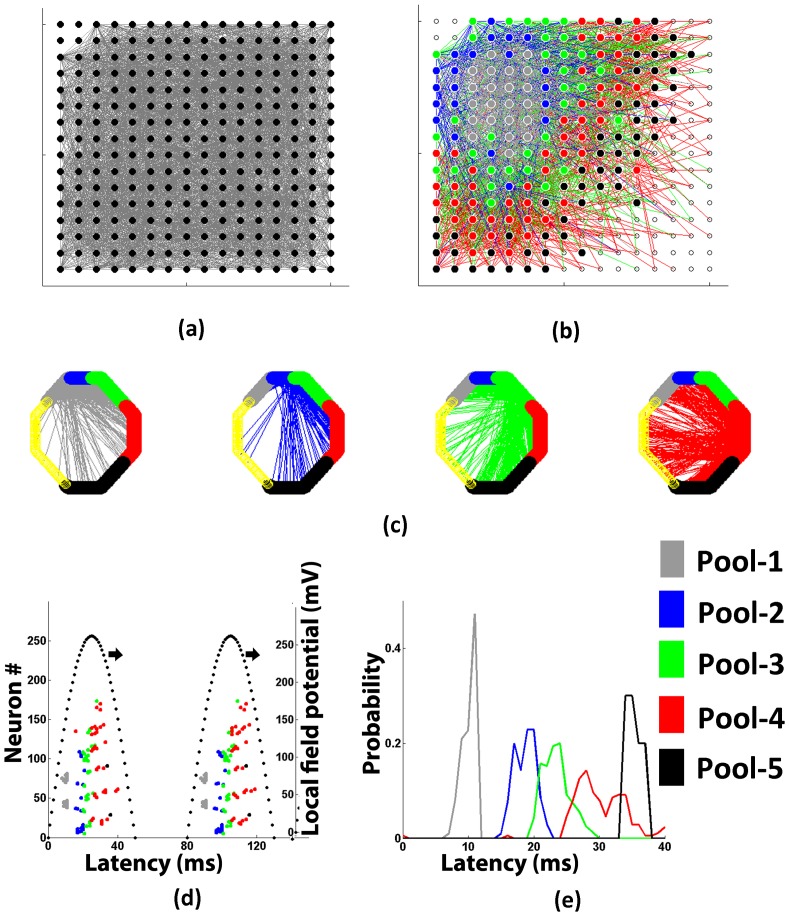
Spontaneously formed Synconset chains predict synconset waves. (**a**) A 252 neuron network under study. Neurons are shown as black circles and synaptic connections are seen as a dense grey haze. This network was created using a neuron distance dependant connection probability which falls exponentially with increasing distance. (**b** A partial network showing neuron pools that belong to the synconset chain predicted from the network in (a). The grey coloured circles were the neurons (pool-1) subjected to external stimulation. The subsequent pools of neurons (coloured blue,green,red and black respectively) were predicted using the procedure explained in “Methods”. Notice that for this network, the neuron pools 2 through 5 are arranged in concentric fashion around the initial stimulation area(pool of grey neurons). The coloured edges show the synapses going out of the neurons in each pool. The grey,blue,green and red edges are the synapses going out of pools 1 to 4 respectively. (**c**) A decomposition of the partial network in (b) that shows the post-synaptic neurons of each neuron pool. The neurons were rearranged so that neurons in the same pool are placed adjacent to each other on the hexagon. Each of the four sub figures show the targets of synapses originating in each pool. Notice that each pool has a high connection density to it's neighbouring neuron pool as compared to the rest of the network. It can also be seen that pools make numerous connections to the preceding as well as succeeding pools thus showing that the network is recurrent. But the activation of the pools shows a feedforward pattern, thus showing the power of this technique to detect feedforward paths in recurrent networks (**d**) Raster plot obtained from simulation of network in (a) using a single compartment model and biophysically realistic conductances. The dotted black line shows the extent of the oscillation. The ticks are coloured to show the pool to which a neuron belongs in the synconset represented in (b). (**e**) Onset time distributions obtained from simulation using biophysically realistic conductances. For each predicted pool of (b), a probability distribution of it's onset times in (d) is plotted by taking the histogram of onset times of the pool neurons accumulated over all cycles. The grey curve is the onset time distribution of the initial set of neurons that were stimulated. The onset time distributions of the subsequent neuron pools are shown in blue, green, red and black respectively. Notice that the medians of the distribution of the successive pools increase monotonically, indicating that the synconset chain predicted in (b) is actually seen to be true in the simulation results in (e).

The initial sub population of the cell assembly (or neuron pool or sub assembly) was the first set of active principal neurons in the cell assembly. We hypothesised that the subsequent activations would be caused by convergent inputs from already active neuron pools onto the other neurons. Neurons with large numbers of active pre-synaptic neurons were expected to be recruited early. Starting from the adjacency matrix of a test network and the initial neuron pool (sub assembly 1), we predicted the next four pools that would be activated in sequence (see “Methods”). It may be noted that this sequence of pools constitutes a feed forward synconset chain. We next simulated a test network built using the same adjacency matrix and biophysically realistic conductances in single compartment neurons. We then verified if the activation sequence predicted by the synconset chains matches with the synconset waves obtained from simulation spike trains (Fig. 2).

**Figure 3:**
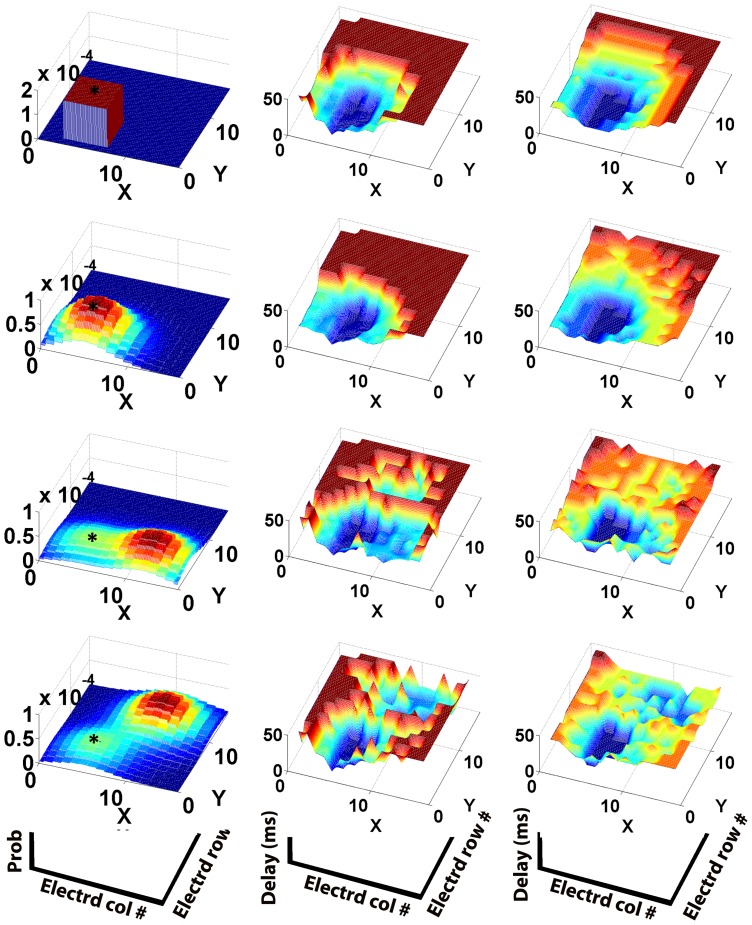
Synconset waves from simulation and prediction mirror the underlying network connection probability distributions. The figures on the left hand side column show the typical probabilities of a synapse from a neuron at the position indicated by the asterisk(‘*’) to the rest of the network. For the networks on rows 1 and 2, the connection probability from any other source to the rest of the network can be obtained by moving the profile such that the asterisk is now at the source to be evaluated. For the network on row 3, connection probability from any source in the left half plane 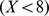 can be obtained by moving the profile as described previously. For sources in right half plane 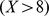, the probability profile at (X,Y) is the mirror image of the profile at 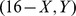 about the line X = 8. This network is an example of the ipsilateral connection scheme commonly found in biological networks. For the network in row 4, probability profile in the quadrant 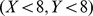 is obtained by moving the probability profile. For sources in the quadrant 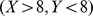 the profile at (X,Y) is given by reflecting the profile at (16-X,Y) about the line X = 8. Sources in the quadrant 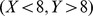 are given by reflection of the profile at (X,16-Y) about Y = 8. Sources in the quadrant 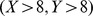 require both the reflections described above (about 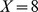 and 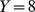). This is an example of the contralateral connection scheme commonly found in biological networks. Bluer shades indicate troughs and hence lower probabilities. Redder shades indicate peaks and hence higher probabilities of connection. The figures on the middle column were obtained from simulations and show the typical onset profiles measured in the corresponding network to the left when stimulation was provided to a subset of 22 neurons around the area indicated by the asterisk. The onset profiles are an interpolated 3-d representation of the onset rasters. Bluer shades are troughs and indicate early firing neurons, with redder shades denote late firing neurons. The earliest firing neurons in dark blue are the stimulated neurons. The right hand side column depicts the predicted onset profile from the adjacency matrix and blue/red shades hold their usual meaning. Notice the relation between the connection probabilities on the left, onset profiles on the middle and right. Areas with high connection probability on the left correspond to early firing areas on the middle and right. The onset profile due to stimulation is a reflection of the connection probability from the stimulated area to the rest of the network. The simulated profiles in the middle are slightly curtailed compared to the predicted profiles on the right due to incomplete activation of downstream neuron pools, though the order of pools is still maintained between simulation and prediction.

The combination of a network and an input stimulation subset comprised a code. The extreme right and centre columns in Fig. 3 show the onset profiles predicted by the synconset chains and the synconset waves obtained from simulation of four different codes. Notice that the synconset waves obtained from simulation closely mirror the predicted profile. At times the two profiles may seem temporally expanded or compressed with respect to each other though the sequence of activations are largely maintained.

In order to quantitatively verify if a simulated profile matches the corresponding prediction, we analysed the latency time distributions (obtained from simulation) of the predicted sequence of neuron pools. If the prediction was correct, we may expect that the distribution median/mode of successive neuron pools will increase monotonically. Or at the least they should not show any decrease in median latencies. To test the distinctness of distributions, we used a non-parametric test of equal medians (Mann-Whitney) on the onset time distributions of successive neuron pools(for details see “Methods”). Each code was tested with 6 different sets of parameters to find the success rate of each code. The success rate correlated with the number of neurons in the predicted pool 2 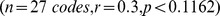, pool 3 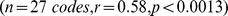, pool 4 
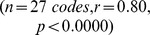
 and pool 5 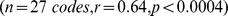. Thus, provided that there are sufficient numbers of neurons in each pool of the synconset chain, the associated synconset wave could be predicted correctly.

### Synconset waves can be used to identify underlying network

Prediction and verification of synconset latency from the network connectivity helps in establishing the legitimacy of onset latency dynamics as an expression of the underlying network connectivity. While this is important from a perspective of understanding the phenomenon, solving the inverse problem lends insights into the underlying network organisation. Hence the next part of our study attempted to gain insights into the network architecture based on synconset latencies.

#### Underlying network can be identified using synconset waves and Bayesian reasoning

**Figure 4:**
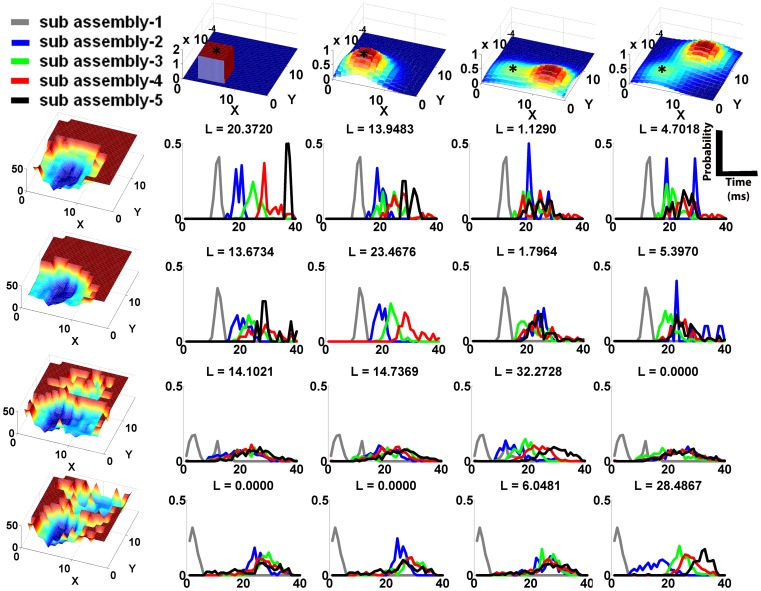
Computing likelihoods of synconsets given a candidate network helps identify underlying network architecture. Each column is associated with the network represented by the connection probability diagram on the top header. Each row is associated with an onset profile shown on the extreme left. The connection probability diagrams and onset profiles are to be read as in Fig.(as “L =  xx.yyyy”). The likelihood value is a measure of the probability that the onset profile on the extreme left was obtained from the network on the top row. Notice that likelihood is highest along the diagonal where the onset profiles intersect with the true network that expressed them. The high value of likelihood comes from the well separated histograms with monotonically increasing medians of different neuron pools. For instance at the intersection of the first row and first column, notice the grey, blue, green and red curves have increasing medians and are well separated compared to the rest of the histograms on row 1. Likelihood values of zero result when the medians of the curves are not in increasing order.

Transforming the synconset profiles directly to a network representation is a very tough problem due to the high dimensions involved. Instead we chose to tackle a slightly less intensive problem, one of identifying the underlying networks from a finite set of candidate networks. Thus we may start with a viable set of candidate networks and identify one that is most likely. We shall defer until the next subsection the problem of constructing this set of candidate networks. For now we remain agnostic to the manner in which this set was constructed and focus on the problem of network identification from a set of candidates. In order to compare multiple possible network reconstructions we need a quantitative measure that indicates the probability of a candidate network given the observed synconset wave. As shown in “Methods”, it turned out that all networks being equally likely, the network that maximises the likelihood of an onset dynamic was also the most probable network for the onset dynamic. As an illustration Fig. 4 shows four synconset waves obtained from different networks being tested against four candidate networks. Notice that the likelihoods are high along the diagonal.

A total of 9 different networks were simulated with 6 different sets of simulation parameters (for details refer “Methods”), thus providing 54 sets of simulations. Three different onset profiles were observed during each of the simulations, by stimulating three different subsets of neurons at different times. Each of these 54 simulation yielded a set of 3 different synconset waves. Using this synconset wave set it was required to identify the network which produced it. As explained in “Methods”, the likelihoods of each of these synconset wave set with respect to all the 9 networks were computed. The network that had the highest likelihood was declared as the identified network from amongst the set of networks. In 50 out of the 54 simulations (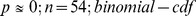), the identified network was the true network. The networks that did not match had very few neurons in their synconset neuron pools and hence were not properly activated.

**Figure 5:**
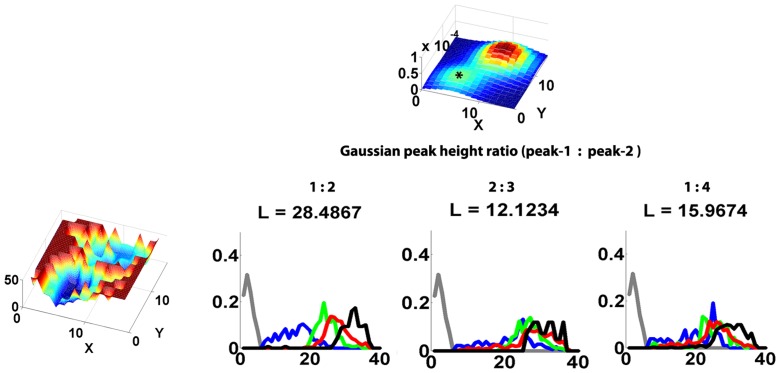
Likelihoods serve as a metric for identifying the closeness of a candidate network to the true network. The histograms are to be read as in Fig.(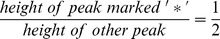). But with similar networks (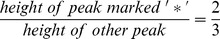 and 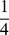) the likelihood values though low, is still higher than that for unrelated networks on the last row of Fig. 4.

The utility of this method depends crucially on how the likelihoods vary within a class of similar networks. For instance, by reconstructing the underlying synconset chains from the synconset wave in row-4, col-2 of Fig. 3 it is possible to guess that the connection probability to some regions in the diagonally opposite quadrant is high. But it is not possible to know the relative probabilities of local connections and connections to the opposite quadrant. Further even if the probabilities are known, many different networks can be constructed to satisfy those rules. Thus likelihood values must be higher for networks of the same class compared to networks of other classes. Our method showed that similar networks have likelihoods that lie close together, and widely different networks have likelihoods that differ by a large amount. For instance in Fig. 5 we see that the likelihood of the onset profile with respect to three different networks of the same class have higher likelihoods than that for other classes, and is highest for the true network. Likelihoods of networks of other classes are zero (last row of Fig. 4) due to lack of monotonic increase in delays.

#### Reconstructing candidate network given observed synconset latencies

**Figure 6:**
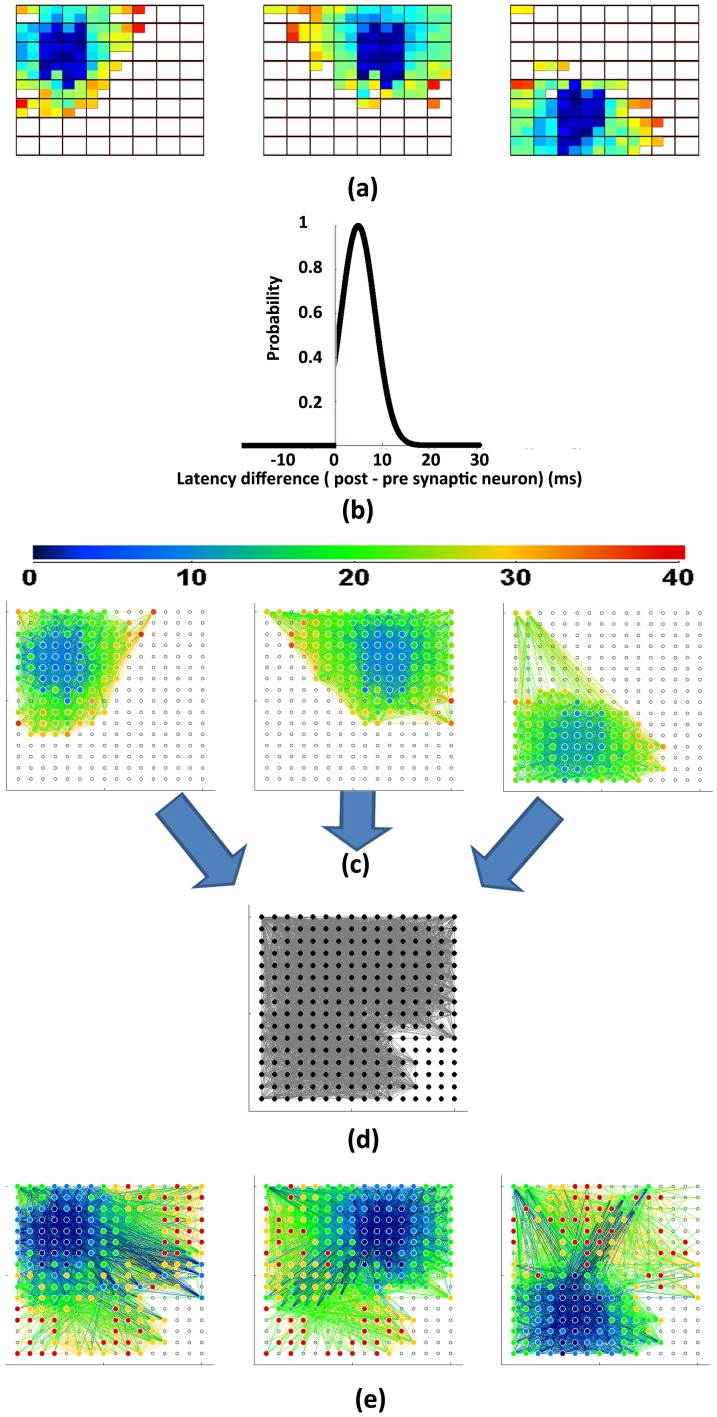
Creating candidate networks from multiple synconset waves. (**a**) The three panels show the observed latencies in response to three different stimuli. Based on the above latency profiles, a reconstruction is to be made. (**b**) A wiring probability to create candidate network. One of the ways of constructing candidate networks is based on the observation that neurons with small difference in their latencies are more likely to be connected. The graph in this figure shows the connection probability of two neurons as a function of their latency difference (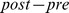). This particular probability scheme always connects a neuron to another one with a higher latency as is evident from the zero probability at negative latency differences. Highest connection probability is to a neuron firing a little later(5 ms) than itself. The probability drops off exponentially on both sides of this maximum. (**c**) Three partial reconstructions of the network corresponding to the three synconsets in (a). The probability profile in (b) was used for this reconstruction. The colours of neurons show the activation latencies as shown in the key. Synapses(edges) take the colours of the pre-synaptic neurons. (**d**) The reconstructed candidate network. This reconstruction is just a merge of the three partial reconstructions in (c). If a synapse exists in any one of the three partial networks in (c), it exists in the merged network too. Notice that this reconstructed network has no edges in the bottom right of the network. This is due to the fact that none of the 3 stimulations of the original network in (a) activated this part of the network. Hence we have no information about it. (**e)** The predicted latency response to the three stimulations in the reconstructed candidate network of (d). Note that the colours now represent the predicted latencies in the new network and not the original latencies as in (a) and (c). Note that these predicted latencies also have a centre out structure as in the original network. Although the detailed structure of latencies is different, the likelihood values for the reconstructed network are of the order of the original network.

From the discussion in the previous sections we know that synconset chains cause first spike cascades or synconset waves. We also know that synconset waves are associated with synconset chains by the simple rule that says “upstream pools fire earlier than downstream ones”. By moving from early firing neuron pools to later firing ones we are in fact moving from upstream pools in the synconset chain to the downstream pools. Using this rule one may intuitively construct the approximate synconset chain for each observed synconset wave by connecting neurons with similar latencies. Fig. 6 shows the reconstruction of three different synconset chains from three synconset waves observed in the same network. In each case the probability of connection between two neurons is a function of the difference between their latencies (Fig. 6b). The three partial networks in Fig. 6c are constructed from the latencies in the three synconset waves of Fig. 6a. After merging the three partial networks, we see that the response to stimuli(Fig. 6e) evokes centre-out responses similar to the original responses (Fig. 6a). But the responses in the reconstructed network differs from the original in their details. For instance, notice the long distance blue synapses from the blue neurons in Fig. 6e which are absent in the original. In our case the aberrations are due to cross coupling between neurons whose latencies are similar. Thus neurons that are activated by a common set fire almost at the same time but do not have any connections in between. Thus it must be emphasised that the particular form of the probability function used in Fig. 6b or the way partial networks are merged are only illustrations of a reconstruction method and no optimality is implied. In fact, whole families of such reconstruction algorithms may be designed by using different probability functions and merging algorithms based on their efficiencies in varying situations. For instance the probability function may be made symmetric to increase connection density to upstream neurons. Increasing the standard deviation of the gaussian leads to faster propagation of synconset waves. Algorithms may also be designed to iteratively refine the reconstruction in order to increase the likelihood value. For instance in this case the long distance blue synapses in 6e may be removed to come up with activations that better resemble the original activation patterns. But the unique feature of our method is that we do not take our reconstructions to be final. The reconstruction is checked for compatibility with the actual latency patterns observed and obtain a measure of the likelihood that this reconstructed network could cause the observed latency patterns. An incompatible reconstruction can be discarded, and a compatible one refined to increase it's likelihood. Robustness of reconstruction can also be tested by stimulating the reconstructed and original network with a new stimulus that was not used during the reconstruction process. In fact within our framework, we can reconstruct networks by any of the traditional methods available in literature. Networks could be reconstructed from anatomical cues or even by guess and intuition. All these reconstructions can be pooled together to form the candidate network set and the probabilities that these networks could have caused the observed synconset waves can be measured.

### Network identification using synconset waves is robust to network sub sampling

**Figure 7:**
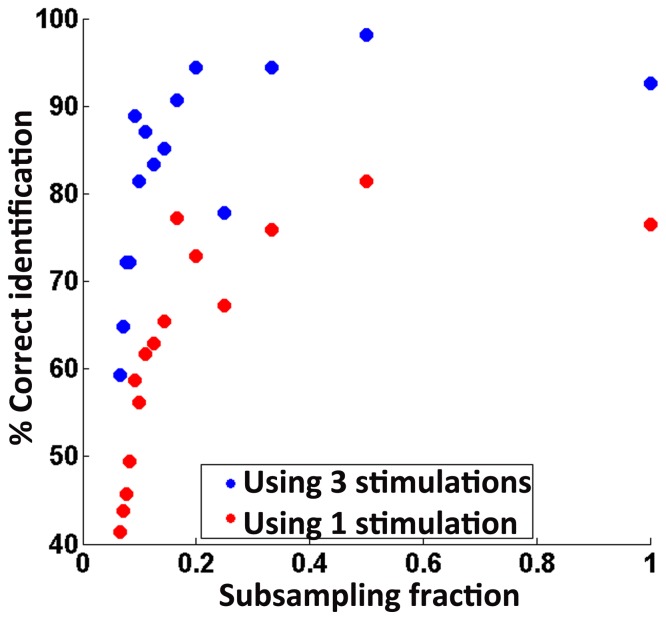
Effect of subsampling on network identification. Figure shows a plot of the number of networks correctly identified (as 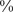 of total) with decreasing subsampling fraction. Red circles correspond to network identification using L-value from a single stimulation. Blue circles correspond to network identification using combined L-value from 3 different stimulations per network. Using more number of synconset waves leads to increased accuracy in network identification. Notice that network identification performance stays in the range of 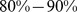 for subsampling ratios as low as 0.1 (1∶10) and declines drastically below that. Subsampling ratio of 0.1 corresponds to a subsampled network that uses information from about 25 out of 252 neurons. Subsampling is done on the grid of neurons and does not use any knowledge of which neurons are actually active during a stimulation. Hence the actual number of neurons used in a 0.1 subsampling is much lesser than 25 (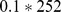) and is of the order of 10 neurons. The critical subsampling ratio below which performance degrades is not a fixed ratio and is dependant on the availability of sufficient number of reliably firing neurons.

A common problem in studying networks of individual neurons is sub-sampling [29], which arises due to the fact that most recordings read only from a fraction of the neurons in the network. The properties of the network measured from a sub sampled reconstruction are not always the same as that of the original network [30], [31]. An important property of onset delays is that the measured delays of a neuron are not influenced by other neurons that were not sampled. Thus, using onset delays are likely to be immune to sub-sampling. To test this, we created sub sampled onset profiles from all the 54 simulations (used in previous section) using various subsampling ratios from 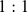 down to 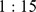 and quantified the network detection performance(Fig. 7). The performance was quantified using 2 methods. The former method used the L-value (equation 15) obtained from a single stimulation of the network to identify the network while the latter used the averaged L-value from 3 different stimulations per network. Network identification using multiple stimulations was more reliable than using a single stimulation. It was also seen (Fig. 7) that network identification performance remained stable until a subsampling ratio of about 0.1 (25 out of 252 neurons). Since these neurons are chosen without regard to which neurons are active during a stimulation, the actual number of active neurons in the sampled set was much below the numbers expected from the subsampling ratio. For instance, using 1∶10 subsampling, the actual numbers of active neurons available are of the order of 10 (instead of 25 as may be expected). It must also be noted that the minimum tolerable subsampling ratio is not a fixed number and depends on the number of reliably firing neurons sampled. In general, as long as there are enough onset delays to compute reasonably smooth delay distributions, the likelihood of candidate networks can be reliably computed. The number of spikes are a product of the number of oscillation cycles observed and the number of neurons observed. Thus even a few reliably firing neurons observed over adequate number of cycles were sufficient.

**Figure 8:**
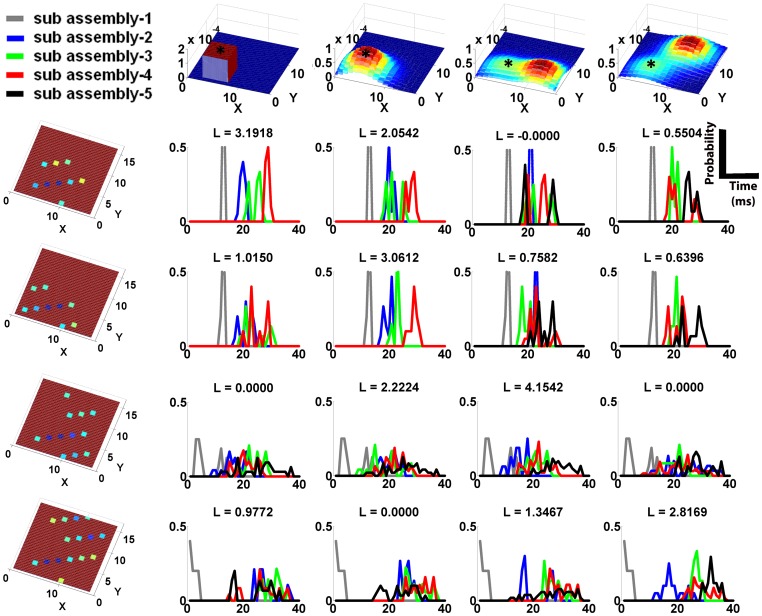
Synconset based network identification works robustly in sub sampled networks. Using onset latencies from only 28 out of the 252 neurons (1∶9 sub sampling), the delay histograms with respect to various candidate networks were constructed as in Fig. 4. The sub sampled onset profiles are indicated on the left extreme and the candidate networks on the top row. Notice that the likelihoods are still high along the diagonal where the sub sampled onset profiles meet true networks. Notice again that along the diagonal the grey, blue,green,red and black curves have monotonically increasing medians and are better separated than the other histograms.

An illustration of 1∶9 subsampled onset profiles and the associated delay distributions for four different profiles and candidate networks are shown (Fig. 8). We see that the different neuron pool delay distributions are still separable. The likelihoods are useful in associating onset patterns with networks as is evident from the dominant L-values on the diagonal.

### Onset sequences and subsequent network identification are robust to variation in simulation parameters and mismatch between prediction and simulation parameters

We wanted to check if the synconset chains and waves were a result of the particular values of our simulation models or prediction parameters. Increasing the excitatory-excitatory synapse gains caused more effective and reliable activation of the synconset neuron pools in simulation and prediction, but the sequence of activations remained the same. Changing the synapse decay time constants to make the synapses slower(or faster) caused the activation sequence to get expanded(or compressed) in time but the sequences were found to be invariant in both prediction and simulation. Extreme values caused a total loss in synchrony (or a simultaneous network-wide onset). For the applicability of our method to data from real networks as against simulation data, it is important that the method can work well when the parameters used in prediction and simulation model parameters did not optimally match. Hence to validate the same, we kept the parameters of the prediction constant, while varying the model parameters in simulation. Our method was insensitive to the exact value of excitatory-excitatory synapse gain within a tolerance band 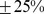 around the mean biophysically realistic value. The prediction was more sensitive to synapse activation and decay time constants and could tolerate deviations of 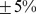 from the mean value. When synapses were slower than the lower end of the tolerance band, the network did not activate at all due to non-synchronous excitation of neuron pools. When synapses were faster than the upper limit of the band, the entire network was activated near simultaneously and hence there were no distinct neuron pools. Synconsets were observed over a range of oscillation frequency from 2–25 Hz. At lower frequencies, the separation between synconsets was clearer and there was sufficient time for the network to deactivate and reactivate again the next cycle. But on the flip side, there were less oscillations and hence fewer onset profiles from which to measure. Higher frequencies had more number of onset profiles but the time between deactivation and subsequent activation shrunk progressively until they overlapped. At this point, the onsets were not clearly visible and hence this method could not be applied. It must be remembered though that the operable range of frequencies is a function of the individual neuron and synapse properties and not an absolute number.

### Scalability of the prediction method with size of network

**Figure 9:**
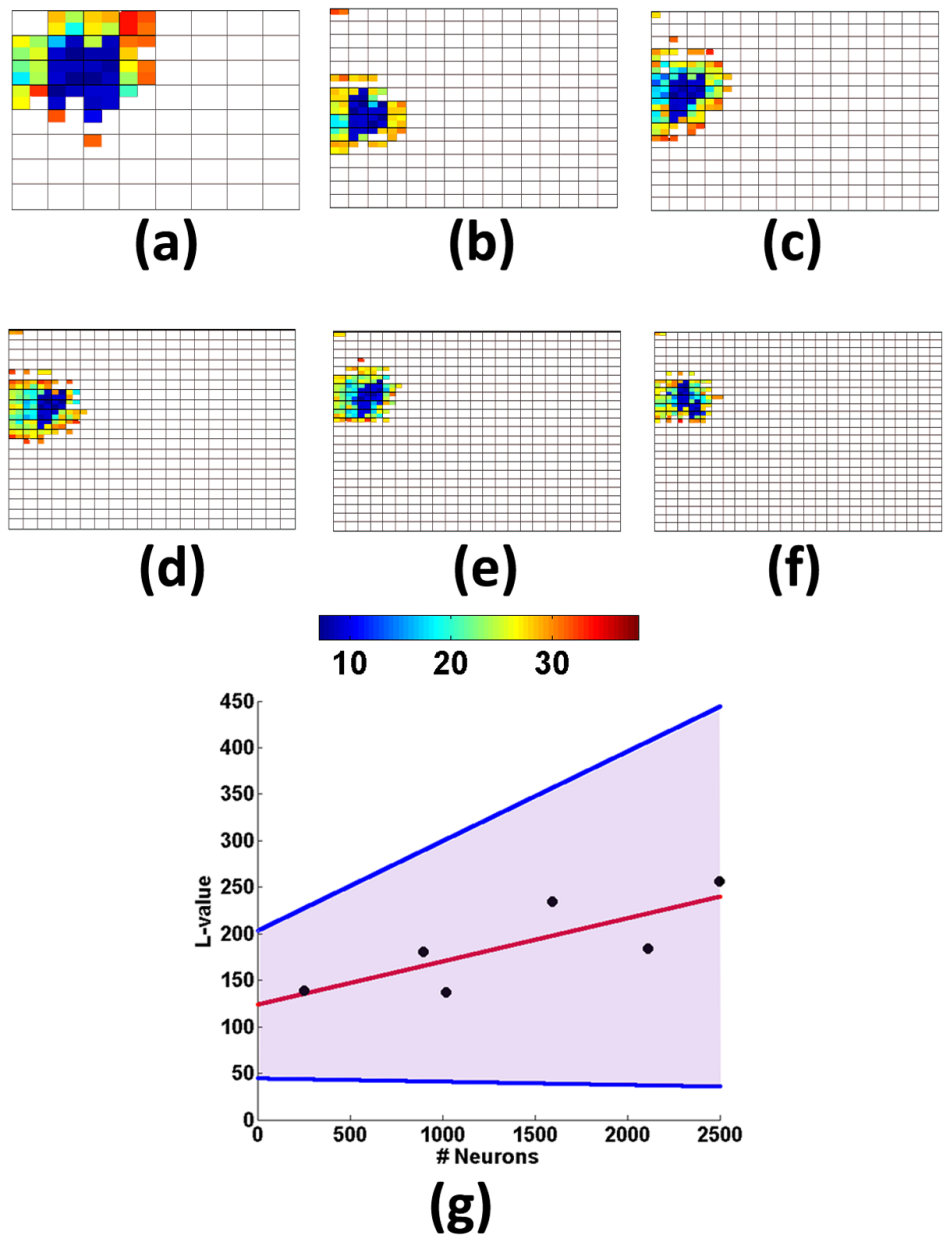
Relationship between network structure and synconset waves holds even when network is scaled (a)–(f) Latency profiles in networks of varying sizes. The 6 networks have all been constructed using the connection probability profile shown in row-2,col-1 of Fig. 3. The sizes of the 6 networks in (a)–(f) are 16X16, 30X30, 32X32, 40X40, 46X46, 50X50 in that order (dimensions in number of neurons). Note that the latency patterns in all the networks are centre-out irrespective of the scale of the network. (**g**) Scatter plot of L-value versus number of neurons in the network. The red line is a least mean-square error fit of the scatter points. The shaded area indicates the region of 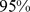 confidence for the regression fit indicated by the red line. Notice that the shaded region is almost entirely in the region of positive slope indicating that the efficacy of the prediction process(L-value) is very unlikely to decrease with increasing network size.

We wanted to further check if the relation between network connectivity and the synconset latencies hold as the network size increases. The most important measure that relates the network connectivity and the synconset latencies is the likelihood (L(D,N)) value of equation 16. This L-value associates a network(N) with the observed synconset latencies(D) in response to a stimulation. It associates the two by specifying a probability that the network(N) was the cause for the effect observed(D). Now if we measured the L-value of association of the true network(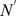) with the actual latencies measured(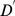) on 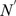 itself, then the L-value observed is actually an indicator of how well the method itself is working. Thus testing the robustness of this L-value with increasing network size should give an idea about the scalability of the method in general. We chose a network with connection probability as in row-2,col-1 of Fig. 3. Keeping the connection probability profile and the average node-degree (synapses per neuron) unaltered, we created networks of 6 different sizes ranging from 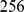 neurons to 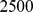. In each of these 6 networks we observed the formation of synconset waves due to three different stimulations. The simulation lengths for all six networks were identical. The L-value (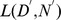) obtained was plotted versus the number of neurons (Fig. 9). We performed a regression on this data to detect any trend in the data. The red line in Fig. 9 shows the fit using a mean square error minimisation. The shaded region shows the 95% confidence interval for the regression straight line. It shows that the L-value does not decrease with increasing number of neurons. Thus the method does not show any degradation with increase in network scale.

## Discussion

### Synfire vs synconset

Spiking onset latency patterns have been observed, classified [5] and analysed in the context of competing clusters in a neuronal culture [32] and network injury detection [7]. But in this work we analyse onset patterns during synchronous firing or “synconset waves” in a more general context and associate them with a structural element that we will call ‘synconset chains’. We use this nomenclature to allude to their similarity to ‘synfire waves’ and ‘synfire chains’. Waves refer to the observed dynamic and chains are structural elements that cause this dynamic. While synfire waves are propagating packets of spikes, synconset waves show cascades in their spiking onsets but not necessarily in all their activity (Fig. 1a–b). Where does this difference come from? Synconset waves, like synfire waves result from chains of feedforward connections. Synfire chains assume that the background input other than from upstream neurons is largely asynchronous or at most mildly correlated with the chain activity and at a comparatively lower rate. But synconset chains make no demands on the rest of the network in it's vicinity (Fig. 1c–d). In fact most of our networks have recurrent feedback structures from downstream neurons back to upstream neurons that could ensure that a neuron once active continues to fire until feedback inhibition shuts them off. In spite of the heavily recurrent network, we are still able to uncover the embedded feed forward chain mainly because recurrent connections may cause sustained firing or bursting of neurons and prevent the formation of packets and synfire waves. But the first activation is still due to the embedded feed forward structure. How then do we uncover the recurrent connections? This can be done by a stimulation that activates the source of the recurrent connection, thereby reversing the roles of the feedforward and recurrent connections. By observing the synconset waves caused by stimulations of different neuron subsets, different parts of the network can be uncovered. The flexibility in the definition of synconset chains ensures that they do not need to be embedded into networks and thus are more likely to occur naturally. Hierarchically the class of synconset chains are a superset of the class of synfire chains. Similarly synfire waves are synconset waves, but not the other way round. In the special case when recurrent connections do not cause repetitive firing due to the intrinsic properties of the neuron itself, synconset waves degenerate into synfire waves. Measuring only the first spike latencies are less demanding than measuring and analysing significant temporal delays from spike trains [33]. But spike latencies are nothing but delays measured at specific time epochs. Thus synconset wave based analysis presents a low complexity analysis of temporal relationships in arbitrary recurrent networks. This study thus brings out the utility of onset latency based analysis and demonstrates it's origins from the network connectivity structure.

### Conditions for expression of synconset waves

The main requirement for a synconset wave is the convergence of near synchronous activity from pre-synaptic neurons at the post-synaptic neuron. This loosely translates into two requirements, namely that the average node degree of the network be above a threshold and that trigger stimulation to the network be given near synchronously to a sufficiently large subset of neurons. The value of the two thresholds mentioned above are linked to the profile of the EPSPs induced in the post-synaptic principal neurons. Smaller EPSPs imply that larger numbers of pre-synaptic activations are required to recruit downstream neurons and larger epsps imply the opposite. For instance, in our simulations, synchronous activations of about 20 pre-synaptic neurons were sufficient to start a synconset wave when the somatic EPSPs were in the higher end of the physiological range of 0.1
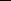
0.5 mV ([34]). But more than 35 pre-synaptic neurons were required when somatic EPSPs were in the lower end of the same range. This effect is also reflected in the fact that successful propagation of synconset waves were positively correlated with the number of neurons activated at each step (neuron pool).

### Synconsets in the context of oscillations, Cell assemblies and population codes

Synconset waves have a special importance in the context of oscillations and synchrony. As is evident onset latency based analysis is significantly less complex than measuring delays between all spikes in spike trains. But this saving comes at a cost. The cost being that now there is a reduction in the available data as all spikes other than first one in a train are thrown away. But the benefits bestowed by oscillations compensate for this disadvantage and make onset latency based analysis a very viable option. An oscillation is the result of the interplay of excitation and balancing inhibition, in conjunction with the intrinsic properties of these and other modulatory neuronal populations [35]–[38]. Since oscillatory activity alternately silences and enables activity in a synchronous population every cycle, there is a corresponding cessation and onset of activity every cycle. Thus the number of observable spiking onsets are highly enhanced due to oscillatory activity. Thus synconset-based analysis is especially relevant in the presence of oscillatory activity. But it comes with a caveat that it can only be applied to a synchronous population sharing the same inhibition or the same graph colour [2].

Cell assemblies are groups of transiently active neurons whose collective near synchronous action can influence the activity of a downstream reader neuron. Such cell assemblies are found at various timescales ranging from a millisecond to a few seconds and can thus be ordered hierarchically to form a “neural sentence” comprising “neural words” [39]. Oscillations with their clear onsets and offsets are natural parsing mechanisms of neural syntax [40]. For instance, multiple gamma oscillations nested within a theta cycle in the hippocampus facilitates multilevel organisation of cell assemblies [41], [42]. In the context of cell assemblies, our work can be interpreted as a synconset based analysis of a single cell assembly modulated jointly by top down inputs and resultant oscillatory activity [10]. The prediction mechanism identifies groups of neurons that start firing at each step and thus represent sub cell assemblies within the cell assembly under study. Thus synconset analysis of a cell assembly can uncover sub cell assemblies.

From a population coding perspective, the initial stimulation subset and the network structure is encoded in the pattern of onset latencies. We thus propose that synconset chains may be a way by which such preferred firing latencies [43], [44] and firing orders [11] are established. Our work may also explain how stimuli which are essentially non-temporal in nature may be coded by latency codes[45]. These onset latencies are visible as significant peaks in the cross correlation histograms of pairs of spike trains and the resultant firing orders[11]. Thus a reader neuron equipped with cellular mechanisms for detecting temporal delays can potentially use this code. The set of viable population codes are determined by the network architecture. For instance if the network architecture is random, stimulating a set of neurons in a local area of the network fails to start a synconset cascade, because the inputs have a very low chance of converging. On the other hand a small world network [46] has a high chance of responding well to such localised excitations due to prevalence of local connections. The necessity of having to respond to local excitations may well be a factor in the ubiquity of small world networks. Complete graphs and scale free networks show global synchronisation and hence the number of sub assemblies may be very few. Nevertheless a complete characterisation of synconset patterns in various graph structures is beyond the scope of this study.

### Synconsets and network reconstruction

Reconstructions of neuronal networks have typically focussed on reconstructing the network on a per synapse basis by thresholding a continuous measure of association between pairs of nodes to yield an adjacency matrix [17]. This approach suffers from multiple lacunae as outlined in the introduction. But more importantly from the perspective of a reader neuron [39], what determines it's all-or-none response is the action of a group of upstream neurons and not single neuron responses. We show how families of methods may be designed to directly reconstruct networks from their onset latency patterns by expressing connection probability between neurons as a function of their latencies. These reconstructions lead to networks that mimic the functional spread of activity in the network. Hence we propose that it could be more useful to reconstruct networks in terms of their functional synconset chains. In particular in our study we mostly use Gaussians to represent the connection probabilities. Experimental studies have found similar patterns in cortical networks, especially in the visual cortex [18], [47]–[50].

A direct transformation of the dynamics into the target network is extremely tough on account of the high dimensionality of the space of networks as well as that of dynamics [2], [17]. Even if achieved, any reconstructed network is only a guess. But it is not possible to know quantitatively, how good an approximation the guess is. Further multiple networks may display a network dynamic. These candidate networks need to be comparatively evaluated. Multiple approaches of network reconstruction will likewise yield different reconstructions, with no way to compare their closeness to the actual network.

In this context our study proposes a combination of the forward and backward approach, where network reconstruction is followed by it's quantitative association with the observed dynamic using a Bayesian approach. This association is done by means of a L-value whose calculation we describe in the section on Methods. The proposed L-value measures the compatibility between the observed latencies and predicted sequence of neuron pools that follows from a knowledge of a network. We thus propose L-value as a measure of association between network dynamics (latencies) and network structure. It must be noted that adding dummy neurons or synapses does not alter the L-value. L-values improve if prediction mirrors the observed latencies. We also show the relative robustness of this metric as a measure of association with increasing network size.

These methods can potentially be extended to an iterative network reconstruction methodology where reconstructed networks are progressively refined while making sure that each refinement leads to an increase in the probability measure that associates the network with the observed dynamic. While we describe some latency based reconstruction methods, our network identification framework allows combination of traditional methods of network reconstruction with latency based reconstructions to form candidate network sets which can be evaluated for compatibility with observed latency patterns.

The use of any network reconstruction strategy in physiology – driven network reconstruction poses peculiar problems due to the sub sampled nature of recordings [30], [31]. In this context we show that even in highly sub sampled networks, the onset profiles can still be reliably associated with various candidate networks as long as the delay distributions are reasonably smooth. The reliability comes from the fact that measured delays between two neurons are not affected by the intervening neurons that were not accessible. Uniform sampling ensures that the delay distributions remain smooth. Degradation in decoding due to non-uniform sampling is graceful. Even in the case of non-uniform sampling, if neurons are sampled in the areas where synconset chains are active, the concerned onset profiles can be decoded. The method is flexible in that the number of neurons sampled can be traded off for increased number of cycles over which measurement is performed. Typically in the visual system, the delay pattern can be stably measured over a time range of 1 second [11]. Thus as frequency of oscillation increases, it is likely that with fewer neurons, the delay distributions will be smooth.

In this study we show that the network structure comprising of synconset chains directly influences the formation of synconset waves in the presence of top-down inputs and the resultant oscillation(similar to PING-Pyramidal Interneuron Gamma). These synconset waves can be used to form testable hypotheses about the underlying network and quantitatively identify the most likely candidate network. We thus show that the utility of synconset chains for network studies, be it population codes, network reconstruction or detection of network disruptions holds a lot of promise.

## Methods

The methods outlined below attempt to capture the relationship between a network (N) and the set of latencies (D) observed in the neurons of this network. Let 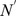 be the network under study. Let us assume that this network is not directly observable and must only be inferred by means of the dynamics of the network. 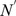 is simulated using NEURON and the voltages of the neurons in the network may be observed. This is akin to observing a neuronal network *in vitro* or *in vivo* with a high density recording device that allows recording at each neuron. The set of spikes of the neurons and their latencies constitutes the actual data (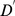) derived from the true network (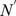). The details of this simulation in NEURON environment is described in the next subsection.

This data set 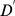 is the only window into the true network 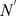. 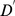 may be transformed to obtain 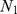, an estimate of 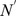. The means for effecting this transform may be one similar to that used in the section “Reconstructing candidate network given observed synconset latencies” or any of the multitude of network reconstruction methods described in literature [17]. Different reconstruction schemes yield various reconstructed networks 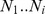.

In order to evaluate a reconstruction 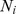 we use a prediction scheme to predict the set of latencies (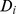) in this network. This is explained in the subsection titled “Prediction of synconset chains from network structure and external stimulation subset”.

In the subsequent subsection titled “Verification of prediction in simulation” we examine the means to verify if the predicted latencies 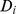 are compatible with the measured latencies 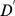.

Finally in the subsection “Computing probabilities of candidate networks given simulation results”, we detail the methods by which we may verify the relative abilities of the various reconstructions 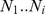 to effect a dynamics such as 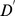. This helps in identifying the network that is most likely to be the true network 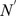 (or it's functional equivalent).

### Simulation and description of models used

Our simulation network consisted of 252 principal neurons and 2 inhibitory neurons placed on a 16X16 grid and was modelled using NEURON. The principal cells were single compartment models with conductance values adapted from [51]. (Peak conductances in 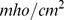: Na,0.015; CaL,0.0025; CaN,0.0025; CaT,0.00057; KAHP,0.0004; Ca activated K,0.00055; KDR,0.009; KA,0.0001; KM,0.00002). Inhibitory cells were Wang Buzsaki cells adapted from [52] (Peak conductances in 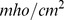: Na,0.035; K,0.009; leak,0.0001). The inhibition was global and acted uniformly on all principal cells. All synapses were exponential synapses (Excitatory : alpha,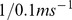; beta,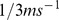 Inhibitory : alpha,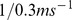; beta,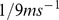). The weights of the excitatory-excitatory synapses were such that EPSPs were in the physiological range of 0.1–0.5 mV (mean = 0.25 mV) [34]. The network was balanced [53] with the standard deviation of membrane voltage within +/−2 mV of the resting membrane potential. In all networks the excitatory-excitatory connection topology were the only ones that changed. All other synapses (inhibitory-inhibitory,inhibitory-excitatory and excitatory-inhibitory) remained exactly the same. A subset of the network (neuron pool-1) comprising 22 neurons was subjected to periodic input stimulation. The stimulation started a cascade of activity in the network, followed by resultant inhibition that silenced the network. This completed one oscillation. Since the input stimulation was periodic, the network activity too was oscillatory. Local field potentials were estimated by summing together the voltage responses of all neurons. Noise was introduced in each neuron independently by injecting external current at random times that caused spikes with probability 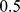. The average firing rate of noise spikes was kept the same as that of input stimulation but their times were uniformly distributed over the entire simulation time.

The combination of a network and an input stimulation set represents a code. We used 9 different networks and 3 different input stimulation sets in each network thus testing 27 different codes. The 3 input subsets were mutually exclusive and covered different regions of the network. Each code was simulated for a total of 2 seconds, roughly the same time over which responses to an input stimulation are sustained in physiological systems *in vivo* [11]. The connection probabilities of the 9 networks are as follows:

- Net1 – As shown in col-1, row-1 of Fig. 3.
- Net2 – As shown in col-1, row-2 of Fig. 3.
- Net3 – As shown in col-1, row-3 of Fig. 3. Ratio of Gaussian peak heights  = 1∶2.
- Net4 – As shown in col-1, row-4 of Fig. 3. Ratio of Gaussian peaks  = 1∶2.
- Net5 – Similar to Net1 with an additional uniform probability centred at it's reflection about y = 8.
- Net6 – Same as Net3, but ratios of Gaussian peak heights are 2∶3.
- Net7 – Same as Net4, but ratios of Gaussian peak heights are 2∶3.
- Net8 – Same as Net4, but ratios of Gaussian peak heights are 1∶4.
- Net9 – Unimodal Gaussian. In the top half, for a pre-synaptic neuron at (x,y) the Gaussian is centred at (x−3,y−3) and centred at (x−3,y+3) in the bottom half.

Each of the 27 codes was tested with 6 different sets of parameters

- Input frequency = 2 Hz, Excitatory synapse gain  = 1X, synapse alpha,beta  = 1X;
- Input frequency = 2 Hz, Excitatory synapse gain  = 1.2X, synapse alpha,beta  = 1X;
- Input frequency = 2 Hz, Excitatory synapse gain  = 0.8X, synapse alpha,beta  = 1X;
- Input frequency = 2 Hz, Excitatory synapse gain  = 1X, synapse alpha,beta  = 0.95X;
- Input frequency = 25 Hz, Excitatory synapse gain  = 1X, synapse alpha,beta  = 1X;
- Input frequency = 25 Hz, Excitatory synapse gain  = 1.2X, synapse alpha,beta  = 1X;

where 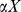 represents 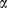 times the chosen biophysically realistic value referred to above.

Each simulation yielded the associated spike rasters and local field potentials. The spike rasters are segmented on a per oscillation cycle basis. One oscillation cycle comprises of a stimulation along with it's consequent inhibition. In each segment, all spikes other than the first spike of a neuron are discarded to obtain one onset profile per cycle.

### Prediction of synconset chains from network structure and external stimulation subset

Given the network adjacency matrix and the initial subset of neurons subjected to stimulation this procedure identifies the sequence of neuron pools that are activated. The following prediction algorithm is run in discrete time in steps of unit synaptic delay as follows.
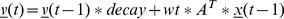
(1)

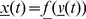
(2)where 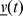 is a vector of neuron voltages at time t,

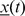
 is a vector of binary numbers indicating if a neuron is spiking or not at time t,
- *A* is the network adjacency matrix,
- *f* is a threshold function which takes a value 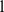 when 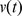 crosses a threshold 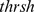 and is zero otherwise,
- *wt* is a scalar representing the epsp amplitude and depends on the synapse gain
- *decay* is a scalar decay constant,
- activations are assumed to be instantaneous,

The onset time of each neuron is the earliest time t when 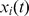 is 1, where 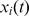 is the 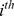 component of the vector 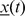. The prediction algorithm is run for 5 time units. The set of neurons with onset time 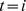 form the neuron pool i. Pool 1 is the set of neurons that were stimulated. The prediction parameters 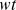,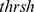 and 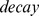 were set such that the network was activated completely and the different neuron pools were distinct. The result of the prediction is a finite sequence of neuron groups that are activated in order. This sequence of neuron pools comprise the synconset chains. Synconset chains implicitly are associated with synconset waves with upstream pools firing earlier than downstream pools.

### Verification of prediction in simulation

Given a predicted neuron pool sequence (synconset chain) and the actual simulation results, it is required to verify if the sequence of synconset neuron pools predicted, matches with simulation. We will initially consider a procedure where this decision will be a binary match/no-match decision (as used in the first subsection of “Results”). In order to use the onset times and verify if it holds in prediction, one needs to determine the demarcating times between the end of activation of a neuron pool and the start of the next. But finding such demarcations in spike data from simulation or real recordings is non trivial and arbitrary. But the predicted data is already grouped into neuron pools and hence we verify if it holds in simulation.

We first grouped together the first spike times in every simulation oscillation cycle from all neurons belonging to the same predicted neuron pool (Fig. 2e). Computing the histogram of onset times in a pool gives the onset time distribution corresponding to the pool. If the prediction matches simulation, the median delay of each of the sequence of pools should increase monotonically. The simulation data was deemed to be compatible with the prediction when medians of consecutive pools were significantly different(Mann-Whitney) and increased monotonically or were insignificant. When the difference between medians of two consecutive pools were statistically insignificant, it meant that two pools that were distinct in prediction have merged together in the simulation. Such differences typically occur due to mismatch of the prediction parameters and the simulation model parameters. We do not treat such merging of neuron pools as an incompatibility as they are inevitable in realistic settings where the neuron and synapse parameters of real neuronal networks are not known and hence do not match. But when consecutive pools have significantly different medians and the medians decrease, it is clearly a violation of the predicted sequence. This verification of prediction in simulated data was performed for all 162 simulations (6 runs each of 27 codes).

### Computing probabilities of candidate networks given simulation results

For the purpose of network identification, binary match/no-match decision is not sufficient. We need to associate a quantitative measure to the probability of different candidate networks given the observed onset latency dynamics embodied by the simulation.

Let N be a candidate network described by it's connectivity matrix.
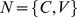
(3)

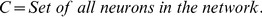
(4)

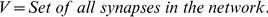
(5)


Let the observed onset latency data be denoted by D. D is thus the set of latencies of all neurons observed during all oscillation cycles.
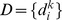
(6)

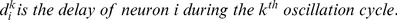
(7)

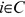
(8)

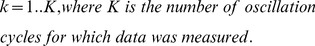
(9)


Now the probability that the network underneath is the candidate N, given that the observed latency data is D, is given by
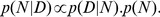
(10)


Assuming that all networks are equally likely, the posterior 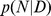 is maximised when the likelihood 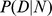 is maximum. Thus in this case, the candidate network with maximum likelihood also maximises the posterior and is the best match network for the observed dynamic. Though we choose a uniform prior for illustration purposes, the methods presented are equally applicable for non-flat priors. For an arbitrary prior distribution instead of comparing the likelihoods directly, they must be weighted by their priors before comparison. These likelihood values 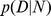 are calculated as explained below. Using the candidate network N the sequence of neuron pools(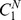 through 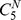) were computed as explained in the section above on prediction of synconset chains. We next grouped together the first spike times in every oscillation cycle from all neurons belonging to the same predicted neuron pool (Fig. 2e).
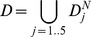
(11)

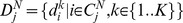
(12)

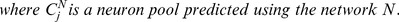
(13)


**Figure 10:**
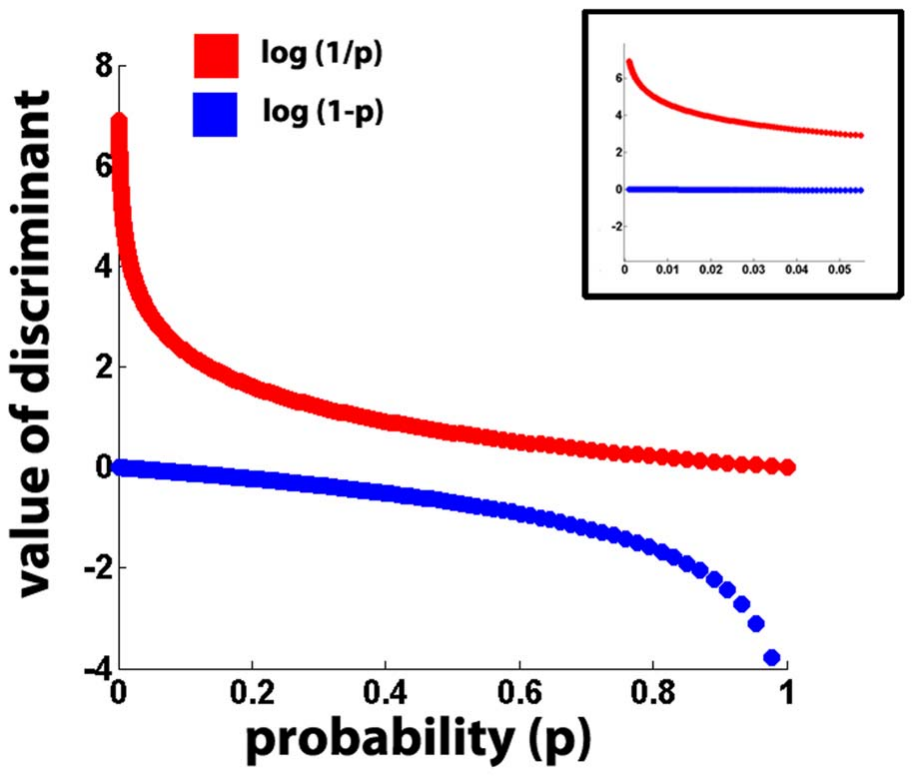
A 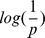 function has better discrimination at lower values of p than 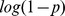. p(probability) value on the x-axis is derived from test of equal medians between two distributions. The blue curve shows a plot of 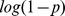 for p-values in the range [0,1]. The red curve shows the same for 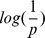. Note that the blue curve is steep at high p values and flat elsewhere, while the red curve is steeper at low values of p and flat later on. Steeper curves imply better discriminability as the value varies by large amounts for even small changes in probability. Inset shows a zoom of the graph in the region of p between 0 and 0.05 which is the area of interest. Note that the blue curve is almost flat and hence unable to differentiate between different values of p. But the red curve becomes steeper as p approaches zero.

**Figure 11:**
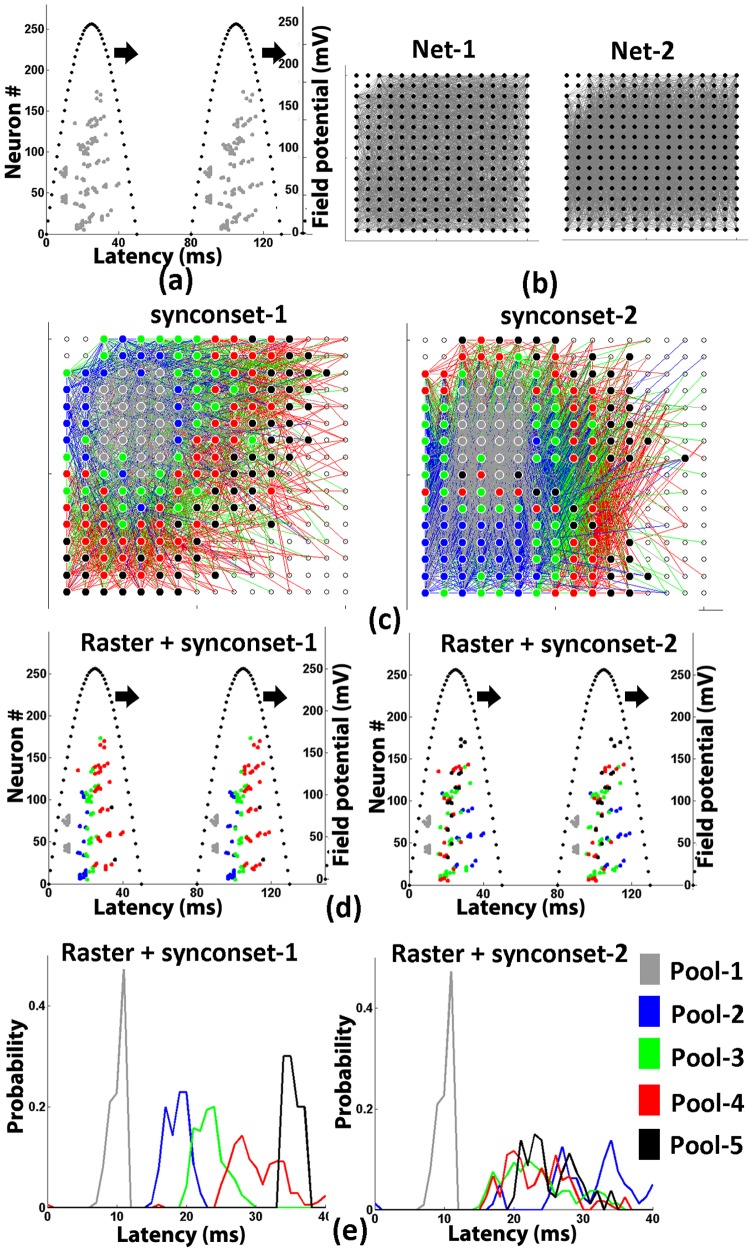
Network identification using synconsets. (**a**) A synconset wave. Grey ticks show first spike times and dotted line shows oscillations. The rasters were obtained from the simulation of the unknown network to be identified. Simulation used single compartment neuron models with biophysically realistic conductances. (**b**) Two candidate networks. Candidate networks may be hypothetical networks constructed from synconsets as explained in Fig. 6 or networks chosen from different classes of networks. (**c**) Predicted synconset chains. The two networks are partial representations of the candidate networks in (b) with the neurons coloured to indicate their membership in various pools. Note that the two predicted synconsets have unchanged pool-1(site of external stimulation) but the compositions of the subsequent pools are different due to differences in network connectivity. The prediction is done as explained in section “Methods”. The coloured edges show the synaptic connections going out of the different pools (**d**)Coloured synconset waves. The two synconsets shown are the same as (a) but with colouring schemes derived from the two predicted synconsets shown in (c). The colours in the two rasters indicate the pool to which a neuron belongs in respective synconset chain in (d). (**e**) Onset time(first spike latency) distributions for the two candidate networks. Onset times from (d) belonging to the same neuron pool over all oscillation cycles are brought together and their histograms are calculated as illustrated in Fig. 2. The histogram distributions of neuron pools with increasing medians indicates that the corresponding candidate network in (b) is a plausible network. When there are multiple plausible networks, the network that yielded a distribution with maximum likelihood is chosen as the identified network. Note in this figure that computing onset time distributions for the raster using synconset-1 yields distributions whose medians increase monotonically for pools 1 through 5. But distributions computed using synconset-2 leads to a loss of monotonicity (median of blue curve(pool-2) is larger than that of other pools). Thus between net-1 and net-2 in (b) net-1 is more likely to be the unknown network.

Computing the histogram of the set of onset times(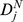) of a pool(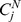) gives the onset time distribution corresponding to the pool. If the distribution of the neuron pool onset times (as in Fig. 2e) yields non overlapping distributions with increasing medians, then the likelihood 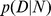 is 1. Needless to say, if even a single pair of consecutive distributions have decreasing medians that are statistically significant, the likelihood must be zero. Interpolating between the two extremes, when the neuron pool sequence is partially overlapping, the likelihood should take a value between zero and one. The value of this probability is defined in terms of the statistics of the onset times.
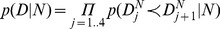
(14)

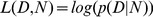
(15)

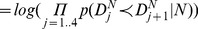
(16)

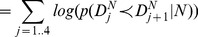
(17)where 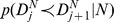 is the probability that the neuron pool 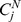 fires earlier than pool 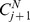 in data D, given the network N. The value of 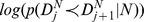 may be defined as 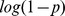, where p is the p-value of the Mann-Whitney test of equal medians between the distributions 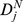 and 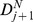. We want the L-values to be as different as possible for mildly differing values of 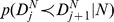. This will enhance the discriminability of different candidate networks. On this count using 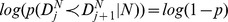 leads to very low discriminability. Using 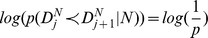 instead, remarkably enhances discriminability(see Fig. 10). But 1/p is greater than 1 and hence not a probability density. Nevertheless we choose to use the latter since our primary purpose is to differentiate between different networks. When the test of equal median returns a 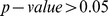 then the distributions have equal medians with high probability and we reset the p-value to 1 in order that it's contribution to L-value may be zero. Similarly when the 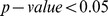 but the median of 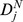 is greater than that of 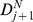, then the pool 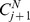 is firing earlier than 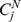 in the dataset D given network N. Here again we reset the p-value to 1. We also assume that the log sum is taken only over consecutive pairs of pools with statistically distinct medians (Mann-Whitney, 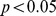). When multiple onset profiles from different stimulations were used, the likelihood values(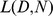) were calculated separately for each stimulation and then averaged to obtain a composite log-probability measure. Figure 11 illustrates the working of this method in order to identify the correct network. Figure 11a shows a first spike raster from an unknown network. The two candidate networks to be tested are shown in Fig. 11b. When the neurons are categorised into groups using the true network, the distribution of latencies line up so the mode/median progresses monotonically from pools 1 through 5 as in the panel on the left in Fig. 11e. The same is not seen when the wrong network is used to categorise neurons as in the right panel of Fig. 11e.

## References

[1] Cardin JA, Carlen M, Meletis K, Knoblich U, Zhang F, et al (2009) Driving fast-spiking cells induces gamma rhythm and controls sensory responses. Nature 459: 663–667. 19396156 10.1038/nature08002 PMC3655711

[2] Assisi C, Stopfer M, Bazhenov M (2011) Using the Structure of Inhibitory Networks to Unravel Mechanisms of Spatiotemporal Patterning. Neuron 69: 373–386. 21262473 10.1016/j.neuron.2010.12.019 PMC3150555

[3] Buzsaki G, Chrobak JJ (1995) Temporal structure in spatially organized neuronal ensembles: a role for interneuronal networks. Current Opinion in Neurobiology 5: 504–510. 7488853 10.1016/0959-4388(95)80012-3

[4] Roelfsema PR, Engel AK, Konig P, Singer W (1997) Visuomotor integration is associated with zero time-lag synchronization among cortical areas. Nature 385: 157–161. 8990118 10.1038/385157a0

[5] Raichman N, Ben-Jacob E (2008) Identifying repeating motifs in the activation of synchronized bursts in cultured neuronal networks. Journal of Neuroscience Methods 170: 96–110. 18281097 10.1016/j.jneumeth.2007.12.020

[6] Srinivas KV, Jain R, Saurav S, Sikdar SK (2007) Small-world network topology of hippocampal neuronal network is lost, in an in vitro glutamate injury model of epilepsy. The European journal of neuroscience 25: 3276–86. 17552996 10.1111/j.1460-9568.2007.05559.x

[7] Raghavan M, Amrutur B, Srinivas K, Sikdar S (2012) A study of epileptogenic network structures in rat hippocampal cultures using first spike latencies during synchronization events. Physical Biology 9: 056002. 22878674 10.1088/1478-3975/9/5/056002

[8] Takano H, McCartney M, Ortinski PI, Yue C, Putt ME, et al (2012) Deterministic and stochastic neuronal contributions to distinct synchronous ca3 network bursts. The Journal of Neuroscience 32: 4743–4754. 22492030 10.1523/JNEUROSCI.4277-11.2012 PMC3328771

[9] Beggs JM, Plenz D (2004) Neuronal Avalanches Are Diverse and Precise Activity Patterns That Are Stable for Many Hours in Cortical Slice Cultures. The Journal of Neuroscience 24: 5216–5229. 15175392 10.1523/JNEUROSCI.0540-04.2004 PMC6729198

[10] Tiesinga P, Fellous JMM, Sejnowski TJJ (2008) Regulation of spike timing in visual cortical circuits. Nature Reviews Neuroscience 9: 97–107. 18200026 10.1038/nrn2315 PMC2868969

[11] Havenith M, Yu S, Biederlack J, Chen N, Singer W, et al (2011) Synchrony makes neurons fire in sequence, and stimulus properties determine who is ahead. The Journal of Neuroscience 31: 8570–8584. 21653861 10.1523/JNEUROSCI.2817-10.2011 PMC6623348

[12] Luczak A, Barthó P, Marguet S, Buzsáki G, Harris K (2007) Sequential structure of neocortical spontaneous activity in vivo. Proceedings of the National Academy of Sciences 104: 347–352. 10.1073/pnas.0605643104 PMC1765463 17185420

[13] Uhlhaas PJ, Pipa G, Lima B, Melloni L, Neuenschwander S, et al (2009) Neural synchrony in cortical networks: history, concept and current status. Frontiers in integrative neuroscience 3: 17. 19668703 10.3389/neuro.07.017.2009 PMC2723047

[14] Herrmann M, Hertz J, Prügel-Bennett A (1995) Analysis of synfire chains. Network: computation in neural systems 6: 403–414.

[15] Aviel Y, Pavlov E, Abeles M, Horn D (2002) Synfire chain in a balanced network. Neurocomputing 44: 285–292.

[16] Trengove C, van Leeuwen C, Diesmann M (2012) High-capacity embedding of synfire chains in a cortical network model. Journal of Computational Neuroscience : 1–25. 10.1007/s10827-012-0413-9 PMC3605496 22878688

[17] Bullmore E, Sporns O (2009) Complex brain networks: graph theoretical analysis of structural and functional systems. Nature Reviews Neuroscience 10: 186–198. 19190637 10.1038/nrn2575

[18] Ko H, Hofer S, Pichler B, Buchanan K, Sjöström P, et al (2011) Functional specificity of local synaptic connections in neocortical networks. Nature 473: 87–91. 21478872 10.1038/nature09880 PMC3089591

[19] Briggman K, Helmstaedter M, Denk W (2011) Wiring specificity in the direction-selectivity circuit of the retina. Nature 471: 183–188. 21390125 10.1038/nature09818

[20] Bock D, Lee W, Kerlin A, Andermann M, Hood G, et al (2011) Network anatomy and in vivo physiology of visual cortical neurons. Nature 471: 177–182. 21390124 10.1038/nature09802 PMC3095821

[21] Chklovskii D, Vitaladevuni S, Scheffer L (2010) Semi-automated reconstruction of neural circuits using electron microscopy. Current opinion in neurobiology 20: 667–675. 20833533 10.1016/j.conb.2010.08.002

[22] Denk W, Briggman KL, Helmstaedter M (2012) Structural neurobiology: missing link to a mechanistic understanding of neural computation. Nature Reviews Neuroscience 13: 351–358. 22353782 10.1038/nrn3169

[23] Csicsvari J, Hirase H, Czurko A (1998) Reliability and state dependence of pyramidal cell interneuron synapses in the hippocampus : an ensemble approach in the behaving rat. Neuron 21: 179–189. 9697862 10.1016/s0896-6273(00)80525-5

[24] Barthó P, Hirase H, Monconduit L, Zugaro M, Harris KD, et al (2004) Characterization of neocortical principal cells and interneurons by network interactions and extracellular features. Journal of neurophysiology 92: 600–608. 15056678 10.1152/jn.01170.2003

[25] Patnaik D, Sastry P, Unnikrishnan K (2008) Inferring neuronal network connectivity from spike data: A temporal data mining approach. Scientific Programming 16: 49–77.

[26] Lee A, Wilson M (2004) A combinatorial method for analyzing sequential firing patterns involving an arbitrary number of neurons based on relative time order. Journal of neurophysiology 92: 2555–2573. 15212425 10.1152/jn.01030.2003

[27] Eytan D, Marom S (2006) Dynamics and effective topology underlying synchronization in networks of cortical neurons. The Journal of neuroscience 26: 8465–8476. 16914671 10.1523/JNEUROSCI.1627-06.2006 PMC6674346

[28] Buzsaki G (2004) Large-scale recording of neuronal ensembles. Nature Neuroscience 7: 446–451. 15114356 10.1038/nn1233

[29] Lee SH, Kim PJ, Jeong H (2006) Statistical properties of sampled networks. Physical Review E 73: 016102. 10.1103/PhysRevE.73.016102 16486211

[30] Gerhard F, Pipa G, Lima B, Neuenschwander S, Gerstner W (2011) Extraction of network topology from multi-electrode recordings: Is there a small-world effect? Frontiers in Computational Neuroscience 5. 10.3389/fncom.2011.00004 PMC3036953 21344015

[31] Stumpf MPH, Wiuf C, May RM (2005) Subnets of scale-free networks are not scale-free: Sampling properties of networks. Proceedings of the National Academy of Sciences of the United States of America 102: 4221–4224. 15767579 10.1073/pnas.0501179102 PMC555505

[32] Shteingart H, Raichman N, Baruchi I, Ben-Jacob E (2010) Wrestling model of the repertoire of activity propagation modes in quadruple neural networks. Frontiers in Computational Neuroscience. 10.3389/fncom.2010.00025 PMC2947946 20890451

[33] Palm G, Aertsen AMHJ, Gerstein GL (1988) On the significance of correlations among neuronal spike trains. Biological Cybernetics 59: 1–11. 3401513 10.1007/BF00336885

[34] Magee JC, Cook EP (2000) Somatic epsp amplitude is independent of synapse location in hippocampal pyramidal neurons. Nature neuroscience 3: 895–903. 10966620 10.1038/78800

[35] Buzsaki G (2009) Rhythms of the Brain. Oxford University Press, USA.

[36] Buzsáki G, Wang X (2012) Mechanisms of gamma oscillations. Annual review of neuroscience 35: 203–225. 10.1146/annurev-neuro-062111-150444 PMC4049541 22443509

[37] Wang X (2010) Neurophysiological and computational principles of cortical rhythms in cognition. Physiological reviews 90: 1195–1268. 20664082 10.1152/physrev.00035.2008 PMC2923921

[38] Atallah BV, Scanziani M (2009) Instantaneous modulation of gamma oscillation frequency by balancing excitation with inhibition. Neuron 62: 566–577. 19477157 10.1016/j.neuron.2009.04.027 PMC2702525

[39] Buzsaki G (2010) Neural syntax: Cell assemblies, synapsembles, and readers. Neuron 68: 362–385. 21040841 10.1016/j.neuron.2010.09.023 PMC3005627

[40] Bartos M, Vida I, Jonas P (2007) Synaptic mechanisms of synchronized gamma oscillations in inhibitory interneuron networks. Nature Reviews Neuroscience 8: 45–56. 17180162 10.1038/nrn2044

[41] Lisman J, Buzsáki G (2008) A neural coding scheme formed by the combined function of gamma and theta oscillations. Schizophrenia bulletin 34: 974–980. 18559405 10.1093/schbul/sbn060 PMC2518638

[42] Sirota A, Montgomery S, Fujisawa S, Isomura Y, Zugaro M, et al (2008) Entrainment of neocortical neurons and gamma oscillations by the hippocampal theta rhythm. Neuron 60: 683–697. 19038224 10.1016/j.neuron.2008.09.014 PMC2640228

[43] Luczak A, Barthó P, Harris KD (2009) Spontaneous events outline the realm of possible sensory responses in neocortical populations. Neuron 62: 413–425. 19447096 10.1016/j.neuron.2009.03.014 PMC2696272

[44] Heil P, Irvine DR (1997) First-spike timing of auditory-nerve fibers and comparison with auditory cortex. Journal of Neurophysiology 78: 2438–2454. 9356395 10.1152/jn.1997.78.5.2438

[45] Panzeri S, Petersen RS, Schultz SR, Lebedev M, Diamond ME (2001) The role of spike timing in the coding of stimulus location in rat somatosensory cortex. Neuron 29: 769–777. 11301035 10.1016/s0896-6273(01)00251-3

[46] Watts DJ, Strogatz SH (1998) Collective dynamics of small-world networks. Nature 393: 440–442. 9623998 10.1038/30918

[47] Bosking W, Zhang Y, Schofield B, Fitzpatrick D (1997) Orientation selectivity and the arrangement of horizontal connections in tree shrew striate cortex. The Journal of Neuroscience 17: 2112–2127. 9045738 10.1523/JNEUROSCI.17-06-02112.1997 PMC6793759

[48] Gilbert C, Das A, Ito M, Kapadia M, Westheimer G (1996) Spatial integration and cortical dynamics. Proceedings of the National Academy of Sciences 93: 615–622. 10.1073/pnas.93.2.615 PMC40100 8570604

[49] Song S, Sjöström P, Reigl M, Nelson S, Chklovskii D (2005) Highly nonrandom features of synaptic connectivity in local cortical circuits. PLoS biology 3: e68. 15737062 10.1371/journal.pbio.0030068 PMC1054880

[50] Ohki K, Chung S, Ch'ng Y, Kara P, Reid R (2005) Functional imaging with cellular resolution reveals precise micro-architecture in visual cortex. Nature 433: 597–603. 15660108 10.1038/nature03274

[51] Migliore M, Cook E, Jaffe D, Turner D, Johnston D (1995) Computer simulations of morphologically reconstructed ca3 hippocampal neurons. Journal of neurophysiology 73: 1157–1168. 7608762 10.1152/jn.1995.73.3.1157

[52] Gloveli T, Dugladze T, Rotstein H, Traub R, Monyer H, et al (2005) Orthogonal arrangement of rhythm-generating microcircuits in the hippocampus. Proceedings of the National Academy of Sciences of the United States of America 102: 13295–13300. 16141320 10.1073/pnas.0506259102 PMC1201613

[53] Chance FS, Abbott L, Reyes AD (2002) Gain modulation from background synaptic input. Neuron35: 773–782. 10.1016/s0896-6273(02)00820-6 12194875

